# Perceptions of medical students towards and effectiveness of online surgical curriculum: a systematic review

**DOI:** 10.1186/s12909-021-03014-x

**Published:** 2021-11-11

**Authors:** Shye-Jao Wu, Ya-Fen Fan, Shen Sun, Chen-Yen Chien, Yih-Jer Wu

**Affiliations:** 1 Division of Cardiovascular Surgery, Department of Surgery, MacKay Memorial Hospital, No.92, Sec 2, Chung-Shan N Road, Taipei, 104 Taiwan; 2 Department of Medicine, MacKay Medical College, New Taipei, Taiwan; 3 Cardiovascular Center, Department of Internal Medicine, Mackay Memorial Hospital, Taipei, Taiwan

**Keywords:** Medical student, Online education, Surgical curriculum, Basic surgical skills

## Abstract

**Background:**

Online education has been used as an adjunct modality for teaching and it attracts attention in recent years as many medical students can not accomplish their clerkship in the hospital due to COVID-19 pandemic. This study aims to collect the articles related to online surgical education for medical students, and to analyze the effectiveness of online education and the perceptions of the medical students.

**Method:**

We performed a systemic literature search in PubMed, MEDLINE, EMBASE, ERIC and Cochrane library. Keywords used for searching included “medical student”, “online education”, “online teaching”, “online learning”, “distance learning”, “electronic learning”, “virtual learning” and “surgical”. Medical education research study quality instrument (MERSQI) was used for the evaluation of the quality of the searched articles.

**Results:**

From 1240 studies retrieved from the databases, 13 articles were included in this study after screening. The publication year was from 2007 to 2021. The mean MERSQI score of the 13 searched articles was 12.5 +/− 1.7 (range 10.0-14.5). There were totally 2023 medical students who attended online surgical curriculum. By online course, improvement of understanding and knowledge on the studied topics could be reached. The confidence in patient encounters could be improved by online curriculum with sharing experiences, discussing, and role playing. However, students felt concentration was poor during online course. Medical students studying through video platform could get better test scores than those studying with textbooks. Regarding basic surgical skills, online teaching of suturing and knot-tying could be possible and was appreciated by the students who could practice away from the hospital and get feedbacks by instructors through online environment. The scores for the clinical competence assessment for incision, suturing and knot-tying were found to be no significant difference between the online teaching group and face-to-face teaching group.

**Conclusion:**

Online surgical curriculum for medical students is not easy but inevitable in the era of COVID-19 pandemic. Although online course is not the same as physical course, there are some efforts which could be tried to increase the effectiveness. Basic surgical skills could also be taught effectively through online platform. Even if the COVID-19 pandemic is over in the future, online curriculum could still be a helpful adjunct for surgical education.

## Introduction

Clinical surgical training is indispensable for medical students. Moreover, in addition to lectures and patient encounters, surgical skills and live demonstration of surgical procedures in the operating room should be very important elements for surgical clerkship. In addition to physical classes, online education has been used as an adjunct modality for teaching in the past. However, due to pandemic of COVID-19, many medical students around the world cannot go through their clerkship in the hospital but complete the training course behind the desk at home by online education [1]. Although it has been reported that the electronic learning could complement traditional teaching methods in undergraduate surgical teaching [2], it is still concerned by the tutors and the medical students whether online education is as much effective as in-person education or not, especially for the training of basic surgical skills. The aim of this study is to collect the published articles related to online surgical education for medical students, to analyze the effectiveness of online education and the perceptions of the medical students and to summarize all the online curriculum designed from all around the world to help medical students to learn what they should learn and what they want to learn.

## Materials and methods

**Fig. 1:**
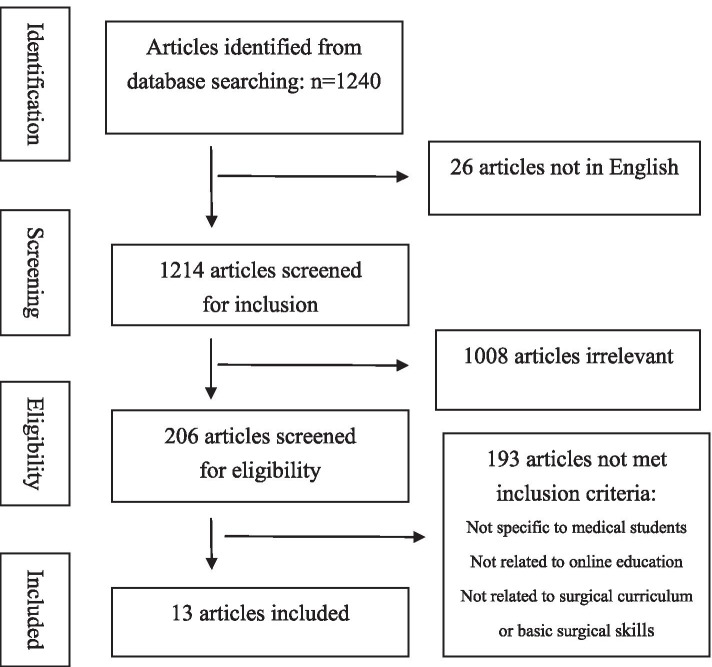
Schematic representation of flow diaphragm for the articles included in the systemic review

This systematic review was conducted in accordance with the guidelines for Systemic Reviews and Meta-analyses [3]. We performed a systematic literature search in PubMed, EMBASE, MEDLINE, and ERIC. There were no similar articles in the Cochrane library. Keywords used for searching included “medical student”, “online education”, “online teaching”, “online learning”, “distance learning”, “electronic learning”, “virtual learning” and “surgical”. The search was limited to the English language and to human studies but not limited to the publication time. In addition to the articles searched by key words, the reference lists of all the relevant articles were also checked carefully. Two investigators independently reviewed the titles and abstracts of the searched articles which were included in this study if the following criteria were met: (1) medical students; (2) online education; (3) curriculum for surgical training. Those articles deemed relevant were selected for further consideration (Fig. 1). Thereafter, data extraction of included studies was completed by two independent reviewers. Only the searched studies related to surgical training for medical students were analyzed [4–16]. The articles not specifically to online education for medical students were not enrolled in this study. The articles related to training of residency were not analyzed. The articles involving teaching of medical students beyond basic surgical skills were not included, either. For the articles reporting the surgical training of both residents and medical students, these articles were discarded if the part of the training of medical students could not be extracted. Review articles were also excluded from this study. The extracted data included author’s name, year of publication, age and gender of medical students, curriculum for online education, effectiveness of the online course, and perceptions of medical students. The extracted data were compiled into Tables 1 and 2.

**Table 1:** Perceptions of medical students for online courses for surgical teaching

Author	Year	St No	St level	Curriculum	Outcomes
Lindeman	2015	129	Grade 2 ~ 4	Blended curriculum: 50% online + 50% in-person (the lectures consistently receiving low student ratings are replaced by online repository of undergraduate-level surgical content)	1.Higher rating for blended course than for traditional teaching 2.No significant difference in national board medical exam or clinical evaluation scores between blended group and traditional group
Shin	2020	16	Grade 3	virtual case-based learning (role playing) 1. students act as doctors 2. instructors act as patients 3. students obtained history gathering, requested PE findings, and brainstormed for DDx	1. confidence in assessment of surgical patients in ER 2. improved content-based knowledge 3. efficacy of stimulating components of clinical work 4. achievement of learning objects 5. recommendation of this course for future students
Chandrasinghe	2020	1047	All grades	Discussion from basic science to clinical management	1.discussion helped to improve understanding and knowledge on the topic 2.improvement in clinical sense through online novel teaching
Kronenfeld	2020	19	Grade 3	1.Electronic education (lectures, PBL) 2.Weekly quizzes	No difference in effectiveness national board medical exam or oral exam between in-person sessions or electronic sessions
Ray	2021	389	All w/o G1	Online education	1. practical training most severely affected by online classes (93.32%) 2. cancelling of physical medical conferences adversely affected the building up of resumes (71.98%) 3. poor concentration during online teaching due to distraction (89.72%) 4. The pandemic has adversely affected the availability of research opportunities and development of skills, ethics, communication and behavior (~ 75%)
Schmitz	2021	44	All grades	Online interactive learning: 1. interactive videos to teach operative procedures and skills 2. information of clinical cases and related excerpts from textbooks	Students taught by video-based online platform got higher score of written exam than by text-based teaching (0.67 vs 0.60, *p* = 0.0001)
Newcomb	2021	5	Grade 4	Virtual class (video watching, role playing, group debriefing, feedbacks from the simulated patient, peer, and faculty)	Improved confidence and skills in building rapport and earning trust of surgical patients by video-conference communication
Pettitt-Schieber	2021	67	Grade 3,4	Virtual surgical teaching (lectures, conferences, suturing, knot-tying)	Improved understanding of surgical subspecialty course (pre 2.0 +/− 0.8 ➔ post 3.3 +/− 0.5 *p* < 0.0001)

*St No* Student number, *w/o* Without, *G* Grade, *PBL* Problem-based learning, *PE* Physical examination, *DDx* differential diagnosis

**Table 2:** Effectiveness of teaching of basic surgical skills for medical students by online courses

Author	Year	St No	St level	Curriculum	Outcomes
Xeroulis	2007	60	Grade 1	Teach suturing and knot-tying with online interactive video demonstration	No significant difference on skill rating between group of interactive video demonstration and group of expert feedback
McGann	2020	86	Grade 3,4	1. Learn to identify instrument, to suture and to tie knots by video instruction 2. Practice by themselves and upload video recording what they did to peers and instructors 3. feedback of instructors by video conference	Nearly all students met their course objectives and significantly increased their confidence
Co	2021	33	Final year	Online demonstration of tutors and online hands-on practice of students for incision, suture and knot-tyingr	No significant difference in global rating scale for technique skills between online group and face-to-face group
Quaranto	2021	31	Grade 3	Interactive remote sessions on surgical instruments, knot tying and suturing techniques	Significant increase in confidence score on knot tying (7.9 +/− 0.7 ➔ 9.7 +/− 0.9, *p* = 0.03) and suturing (8.0 +/− 1.3 ➔ 13.8 +/− 0.9, *p* < 0.001)
Handaya	2021	89	NA	Tutorial video combined with online class for teaching knot-tying	1.pre-online class: the students watching pre-class video got higher score for knot tying (10.9 +/− 1.8 vs 7.5 + /− 1.7 *p* = 0.000) 2.post-online class: no significant difference of scores for tying (11.2 +/− 1.5 vs 10.9 +/− 2.0 *p* = 0.706)

*St No* Student number

Medical education research study quality instrument (MERSQI) was used for the evaluation of the quality of the searched articles. The MERSQI [17] includes 10 items in 6 domains: study design, sampling, type of data, validity, data analysis and outcomes. The maximal score for each domain was 3 and the range of the maximal MERSQI score was 5-18.

Continuous variables were presented as mean +/− standard deviations or median (range or interquartile range (IQR)). Categorical variables were expressed as numbers and percentages. Perceptions of medical students were presented with Likert scale which ranged from strongly disagree to strongly agree.

## Results

By our searching strategy, 1240 studies were retrieved from the databases. After screening, 13 articles were included in this study (Fig. 1).

Among the 13 articles searched from the databases, there were totally 2023 medical students who attended undergraduate surgical curriculum which were composed of online course (1894 students) and combined physical and online course (129 students) and the publication year was from 2007 to 2021. There were 2 studies with all-grade students, 3 studies with grade 3 students, 2 studies with grade 3 and 4 students, and 1 study each with grade 1 students, grade 4 students, final-year students, students except grade 1, students registered to the surgery clinical rotation and students participating longitudinal study design. Among all the 13 searched articles, there were only 3 articles in which age of medical students was mentioned (age range 21-24 years old in 1, median age 23 years old (range 22-24) in 1 and media age 25 years old (range 24-27) in 1), and there were 4 articles in which percentage of female gender was described (45, 47.4, 50.6, 75%,).

There were 8 studies investigating the perceptions of medical students on online surgical education and 5 articles in which the effect of online teaching of basic surgical skills (suture, knot-tying) was studied. The mean MERSQI score of the 13 searched articles was 12.5 +/− 1.7 (range 10.0-14.5).

### Merits of online teaching for medical knowledge and patient encounters

The perceptions of medical students after online surgical education reflected what they needed and what they wanted. Online teaching is not the same way of teaching as the traditionally face-to-face teaching but it is no doubt that the teachers did teach the medical students irrespective of through online platform or in the classroom. In a study by Pettitt-Schieber et al. [15], there were significant increase in understanding of the surgical subspecialty course after online education (pre-course 2.0 +/− 0.8 vs post-course 3.3 +/− 0.5 by 4-point Likert scale, *p* < 0.0001). In a study by Chandrasinghe et al. [5], the medical students felt to be benefited from the discussions during online course, which helped to improve understanding and knowledge on the studied topic and was ranked as 4.9 +/− 0.4 (95% confidence interval 4.8-4.9) by 1046 medical students with 5-point Likert scale [4]. In a study by Newcomb et al. [9], the confidence of medical students in exploring patient’s perceptions, sharing information with patients, checking understanding of patients, exploring concerns, and establishing plans for patients could get improved after virtual classes in which instructors shared their practices for communication with patients by video, followed by a discussion on the students’ former experience of the communication with patients, and then participating role play sessions with simulated patients [9]. There were 80% of the participating students giving “A+” to this curriculum which was not only to teach clinical approaches to surgical patients, but also teach telemedicine through online platform [9]. In a study by Shin et al. [4], the confidence in ability to independently see and complete initial assessment of surgical patients could get improved after virtual case-based curriculum and was ranked as 2.0 (pre-course) to 4.0(post-course) with 5-point Likert scale. For the virtual case-based curriculum [4], medical students were provided with a patient scenario, the instructor leading the discussion role-played as the patient from whom the students obtained history gathering and requested focused physical exam findings and then the students brainstormed for differential diagnoses and diagnostic workup. Moreover, in addition to increasing confidence of medical students on patient encounters, the test score for the content-based knowledge could also be improved [4] from 55% +/− 3.54% (pre-course) to 72.5% +/− 2.14% (post-course) with *p* value 0.0002. In a study by Schmitz et al. [8], medical students studying online with video platform got better test scores than those studying with textbooks (correct rate 0.67 +/− 0.02 vs 0.60 +/− 0.02, *p* = 0.0001). In a study by Kronenfeld et al. [7], there were no significant difference of effectiveness to prepare for national board medical examination and oral examination between in-person sessions and electronic sessions irrespective of faculty-led lectures or resident-led problem based learning. In a study by Lindeman et al. [10], blended curriculum (online teaching plus in-person lectures) was used as a mixed teaching model for basic surgical clerkship and was found to be superior to the didactic lectures. The blended course and in-person teaching was ranked as 3.80 vs 3.52 respectively with 5-point Likert scale (*p* = 0.02) for the lecture series, 4.30 vs 3.98 (*p* = 0.004) for the teaching effectiveness, 3.67 vs 3.36 (*p* = 0.01) for the overall clerkship, and 3.39 vs 3.08 (*p* = 0.03) for the improved confidence.

### Drawbacks of online teaching: distraction

However, online surgical curriculum still had some negative impacts for medical students. In a study by Ray et al. [6], medical students felt concentration was poor during online course. Online class adversely affected the overall learning of which practical learning was adversely affected further [6]. Regarding the role of medical conference on medical education, cancellation of physical medical conference was thought to be adversely affect building-up of medical knowledge, and virtual medical conference was not thought to be an effective tool to enhance learning for medical students [6]. Use of simulated patients and simulation technology for online class were not thought to be useful by medical students [6].

### Teaching of basic surgical skills through online platform

Among the clinical surgical training, in addition to lectures and patient encounters, training of surgical basic skills (suturing, knot-tying) is also one of the important things medical students care most. In a study by Quaranto et al. [14], medical students could increase their confidence score in suturing (pre-course 8.0 +/− 1.3 vs post-course 13.8 +/− 0.9, *p* < 0.001) and knot tying (pre-course 7.9 +/− 0.7 vs post-course 9.7 +/− 0.9, *p* = 0.03) through online teaching. In a study by Xeroulis et al. [11], medical students were asked to learn through online interactive video demonstration and instruction, and then practiced the technique for suturing and knot-tying. There was no significant difference on average global rating scores for basic surgical skills either by interactive video-based instruction or by instruction-only video (without interactive video) and expert feedback [11]. In a study by Handaya et al. [16], the medical students watching the video for knot tying got better scores for knot tying than the students not watching the video (10.9 +/− 1.8 vs 7.5 +/− 1.7, *p* = 0.000) before online classes. After online teaching, irrespective of watching the video for knot tying or not, there was no significant difference in scores of knot tying between the two groups (11.2 +/− 1.5 vs 10.9 +/− 2.0, *p* = 0.706). In a study by McGann et al. [12], e-learning programs with instrument identification, knot-tying and suturing were used as the tools to teach basic surgical skills for medical students who had suture instruments provided in advance and could practice by themselves at home at their paces. Online feedback was given by instructors after the students completed the e-learning and self practice. Regarding instrument identification, knot-tying and suturing, the medical students felt positively about the ability to learn (ranked as 3.4 +/− 1.2, 3.0+/− 1.4, 3.3+/− 1.3 with 5-point Likert scale), the ability of the instructor to teach (ranked as 3.2 +/− 1.2, 3.0+/− 1.3, 3.2+/− 1.3 with 5-point Likert scale), the ability to interact with the instructor (ranked as 3.3 +/− 1.2, 3.4+/− 1.1, 3.7+/− 1.1 with 5-point Likert scale) and the ability to interact with peers (ranked as 3.4 +/− 1.2, 3.5+/− 1.2, 3.7+/− 1.0 with 5-point Likert scale) [12]. In a study by Co et al. [13], web-based learning of surgical skills was used for online teaching for the medical students who had suture instruments given in advance and were taught by the demonstration of the tutors through online environment. The scores for the clinical competence assessment for incision, suturing and knot-tying were found to be no significant difference (*p* = 1.0) between the online teaching group (4.7/5) and face-to-face teaching group (4.8/5) [13].

## Discussion

Online education has been used as an adjunct modality for teaching and it attracts attention in recent years as many medical students can not accomplish their clerkship in the hospital due to COVID-19 pandemic. The way of online learning for medical students is quite different from that of traditional teaching because students are far away from the hospital and the patients. There were many medical students (*n* = 6069) who did not like online teaching and feel online teaching is not effective enough to offer what medical students need [18, 19]. However, online curriculum is inevitable for medical education in the era of COVID-19 pandemic. Moreover, regarding surgical training for medical students, in addition to patient encounters, training of basic surgical skills is another important issue. For the reasons mentioned above, it is more challenging to teach medical students online for surgical training course.

It is known that there are limitations in online education itself when it comes to the efficacy of teaching. Although there were medical students who were not satisfied with online teaching [6, 17], they did still think the instructors were well prepared for the online teaching, which means the students could feel the efforts the instructors have made for the online education [17].

The drawbacks of online teaching mentioned above are not absolutely unable to be overcome. Emphasis on interactive discussions during online teaching could be more stimulating. Instructors usually ask students questions or answer questions requested by students during teaching irrespective of online classes or physical classes. If the students are not familiar with the contents the instructors teach, they usually could not respond fast or adequately and the tuition might be not very impressive for the students. However, if the students are given some time for brainstorming or answer-searching after the questions are asked by the instructors, the whole tuition may become more stimulating and the students may feel they could engage more in the online classes [4]. The break between question asked by instructors and answer responded by students could be tried to achieve better teaching effectiveness and satisfaction.

On the contrary, there were medical students who did express they benefited from virtual class and discussions [5, 9, 15]. Even though medical students did not have real patient encounters, they still could have the feeling of increased confidence in seeing and evaluating surgical patients independently after online course [5, 9], which shows that in addition to didactic lectures, discussion during online education is one of important elements to improve the effectiveness of teaching, and means that it is possible to get acceptable satisfaction from medical students receiving online surgical clinical training.

When it comes to medical conferences, online meetings take place more frequently than before in the era of COVID-19 pandemic. In fact, medical students could experience all the materials presented in the conference including speaker’s presentation and Q/A section irrespective of physical or online conferences. Thus, the effectiveness of online conferences could be similar to that of physical conferences. However, medical students felt the concentration was poor during online teaching, which may be caused by powerful function of computers and electronic devices by which students could do other irrelevant things during online tuition. Distraction during online tuition may have strong impacts on decreasing the merits of online education and ought to be one of the important causes why medical students felt online conference adversely affected the effectiveness of learning.

Regarding teaching with simulation, there were inconsistent opinions expressed by medical students who thought simulated teaching should be a part of medical curriculum but felt simulated patients and simulation technology were not useful for online classes [6]. It has been well known that simulation is one of the important teaching modalities to help medical students approach the real patients in the future, especially for basic clinical skills such as wound suturing, knot-tying, placement of nasogastric tube or urinary catheter, and so on. The inconsistent answers by medical students may imply they felt they could learn and benefit by simulated teaching but patient encounters and learning by doing are what they want the most. Learning by doing is the most direct way to teach medical students how to suture and how to tie a knot and to let medical students be familiar with basic clinical skills, but many medical students lost their opportunities for patient encounters in the era of COVID-19 pandemic and therefore, they could only practice basic surgical skills on the simulated kits but not on the real patients.

In this study, it was found that even by online teaching, medical students still could get satisfactory training for basic surgical skills if they were given suture instruments in advance, was taught with video, practiced suturing and knot-tying with suture kits by themselves, and interacted with online platform or expert feedback [11–14, 16]. Although medical students could get confidence in the ability of basic surgical skills by online classes, they eventually have to practice successfully for the real patients to verify their abilities.

Individualized learning pace could be one of the unique advantages derived from online courses. Students can reach the platform of e-learning at any time and can repeat to approach the online programs as their wishes.

With online course only, medical students may feel they are not well prepared for clinical work in the future. It could be understood why medical students have such kind of viewpoints in mind because they do not see the patients in person during online course and they do not prove their abilities for clinical work by themselves. The feeling of not well preparedness by medical students is really one of the major drawbacks of online teaching but it could be solved and overcome. There are still some methods to improve the effectiveness of online course such as photo-assisted teaching, video-assisted teaching, live demonstration through online platform and so on. In online classes, frequent use of photos and videos may be helpful to let medical students feel they attend patient care or surgeries on-site, which may be able to decrease the students’ depression from cancellation of physical conferences and activities. To the best of our knowledge, this is the first paper of systematic review for online surgical education for medical students.

## Conclusion

Online teaching is not easy but is inevitable in the era of COVID-19 pandemic, especially for surgical training of medical students. Although online course is not the same as physical course, there are some efforts which could be tried to increase the effectiveness of online teaching and to earn the satisfaction from medical students. It is possible to teach basic surgical skills effectively through online platform. Even if the COVID-19 pandemic is over in the future, online curriculum could still be a helpful adjunct for surgical education.

## Data Availability

The data and material will be available on request.

## Abbreviations

COVID-19: Coronavirus disease of 2019
MERSQI: Medical education research study quality instrument
